# Quantifying the Release of Titanium From the Titanium Dioxide-Impregnated Composites Used in Orthodontic Bonding

**DOI:** 10.7759/cureus.42309

**Published:** 2023-07-22

**Authors:** Abhishek Sinha, Sweta Gupta, Taruna Taruna, Leena Priya, Awanindra K Jha, Amesh Golwara, Navmi R Gore

**Affiliations:** 1 Department of Dentistry, Patna Medical College and Hospital, Patna, IND; 2 Departmental of Orthodontics and Dentofacial Orthopedics, Patna Dental College and Hospital, Patna, IND; 3 Department of Public Health Dentistry, Patna Medical College and Hospital, Patna, IND; 4 Department of Oral Medicine and Radiology, Patna Medical College and Hospital, Patna, IND; 5 Department of Orthodontics and Dentofacial Orthopaedics, Dental College, Rajendra Institute of Medical Sciences (RIMS), Ranchi, IND; 6 Department of Dentistry, Dr. Vasantrao Pawar Medical College and Research Center, Nashik, IND

**Keywords:** titanium dioxide, orthodontic brackets, nanoparticles, coupled plasma atomic emission spectroscopy, artificial saliva

## Abstract

Background: Previous literature data has extensively assessed the biocompatibility of various orthodontic adhesives and their components, where the results of most of the studies showed cytotoxic effects of different degrees owing to the unbound molecules released structurally from the cured components.

Aim: The present in-vitro study was aimed to assess the release of titanium dioxide nanoparticles in the artificial saliva from the orthodontic composites impregnated with titanium dioxide nanoparticles of 5% w/w (weight/weight) and 1% w/w used for metal brackets bonding.

Methods: The study assessed 160 teeth extracted freshly during orthodontic treatment and divided into two groups of 80 samples, each that bonded to orthodontic brackets having 5% w/w and 1% w/w composites with titanium dioxide nanoparticles kept in the artificial saliva. Quantification was done for 5% w/w and 1% w/w composites having titanium nanoparticles with inductively coupled plasma mass spectroscopy at 24 hours, two, four, and six months.

Results: It was seen that in teeth with 1% titanium dioxide, the greatest titanium release was seen at two months, with non-significant release after two months. In teeth with 5% w/w titanium dioxide nanoparticles showed significant titanium release all the time. A significantly greater titanium dioxide release on increasing concentration from 1% to 5% was seen for the 5% w/w group at all the assessment times.

Conclusion: The present study concludes that a higher release of titanium is seen in 5% w/w composite containing titanium dioxide nanoparticles, and the concentrations of 1% and 5% can be safely used and are considered to be within permissible limits.

## Introduction

Dental caries result from an interaction between the diet and microorganisms, with plaque being the main etiologic factor. It is crucial to keep in mind that patients receiving fixed or removable orthodontic treatment tend to retain plaque more, which increases bacterial counts [1]. Additionally, it might be difficult to maintain good oral hygiene with orthodontic appliances like brackets and bands that restrict the musculature and salivary mechanical self-cleansing, which raises the risk of developing white spot lesions.

However, various preventive measures were designed that warrant proper and strict patient compliance, including interdental aids, mouthwashes, and oral prophylaxis [2].

Buonocore, in 1955, was the first to introduce acid etch bonding. Since its introduction, various adhesive agents, including resin-modified GIC (glass ionomer cement), compomer, and composites, have been used as bonding agents for fixed orthodontic attachments to the teeth. Composites used in orthodontic bonding have various advantages, including improved esthetics, decreased gingival irritation, good adhesion, and easy handling [3]. The aggregation of bacteria on the adhesive agents is higher owing to its rough surface texture. One method for preventing enamel demineralization and microbial adherence during orthodontic treatment is the use of nanoparticles, which have improved mechanical and antibacterial capabilities [4].

The use of nanotechnology in the dental field has emerged greatly in the past few years, working on the principle of using individual molecules and atoms to form functional structures. Nanoparticles are intentionally produced particles having peculiar dimensions ranging from 1 to 100 nm along with properties shared by the particles of nanoscale with identical chemical composition. Nanoparticles of silica, titanium, zinc, and silver have been used in composites to improve their antibacterial properties [5]. The composites having silver nanoparticles pose superior antibacterial properties with no compromise in the shear bond strength. However, they have shortcomings concerning biocompatibility and composite matrix discoloration. Zinc and copper-based nanoparticles also resulted in toxic effects in the in-vitro studies done in animals [6].

Recently, comprehensive attention has been given to metal nanoparticles made of titanium dioxide owing to their low toxicity and photocatalytic activity. Previous literature data have reported the presence of small pores in bacterial cell walls with the titanium dioxide nanoparticles causing an increase in permeability and cell death that can further prevent enamel demineralization and dental caries. Also, there is very little probability that bacteria will have resistance against titanium dioxide. The mechanical properties of the dental composite material, such as the flexural strength, microhardness, and elastic modulus, as well as the bond strength values higher than or equivalent to the free controls of nanoparticles, can be improved by adding titanium dioxide nanoparticles [7].

To quantify and measure the release of titanium from composites, various methods have been utilized, including cold vapor generation atomic absorption, graphite furnace atomic absorption, flame atomic absorption, flame atomic emission, inductively coupled plasma atomic emission spectrometry, and inductively coupled plasma mass spectrometry (ICP-MS). ICP-MS has various advantages over other techniques, including multi-element capability allowing multiple elements to be assessed at a single time and during a single analysis. Also, ICP-MS is a simple sample presentation and has a short time for analysis, making it a technique of choice [8].

In addition, the biocompatibility of various orthodontic adhesives and their constituents has been assessed in the previous literature data, and the majority of the studies have shown cytotoxic effects of different degrees from unbound molecules released from the cured composite structures [9]. Hence, the present study aimed to quantitively assess the release of titanium dioxide nanoparticles in the artificial saliva from the orthodontic composites impregnated with titanium dioxide nanoparticles of 5% w/w (weight/weight) and 1% w/w used for metal brackets bonding.

## Materials and methods

The present in-vitro study was aimed to quantitively assess the release of titanium dioxide nanoparticles in the artificial saliva from the orthodontic composites impregnated with titanium dioxide nanoparticles of 5% w/w and 1% w/w used for metal brackets bonding. The study samples were processed at the Department of Orthodontics and Dentofacial Orthopedics of the institute after ethical clearance was taken to proceed with the study [PMCH/2023/93].

The inclusion criteria for the study were non-carious and well-defined premolars extracted for orthodontic purpose. The exclusion criteria were hypoplastic teeth, fluorosis-affected teeth, fractured crowns, enamel caries, and teeth having various restorations. The teeth selected for the study were cleaned to remove any tissue debris or blood and were kept in distilled water to prevent any desiccation. 

The study sample comprised 160 freshly extracted premolars for the orthodontic treatment purpose that were divided into two groups having 80 teeth each. The subjects were randomized using computer-generated numbers. The selected teeth were bonded to the orthodontic brackets having 5% w/w titanium dioxide nanoparticles and 1% w/w titanium dioxide nanoparticles forming Groups I and II, respectively. The quantification for the two groups was done at four intervals, including 24 hours, two, four, and six months. The study included anatase phase spherical titanium dioxide nanoparticles in size of 20 to 30 nm owing to decreased photoactivity and surface area.

To formulate nanocomposites of 5% w/w and 1% w/w, 200 mg and 40 mg of titanium dioxide nanoparticles were, respectively, added to 4000 mg of orthodontic adhesives and blended in a composite mixer for five minutes at 3500 revolutions per minute. A scanning electron microscope (SEM) assessment was done for newly formed nanocomposites to evaluate the uniform distribution of composite paste. For bonding, the enamel surface was etched and polished with pumice, followed by drying. The enamel surface was then applied with a primer, and the stainless-steel orthodontic bracket was then bonded, followed by curing for 20 seconds.

The teeth were then placed in artificial saliva having mucin, potassium phosphate, potassium chloride, calcium chloride, and sodium chloride dissolved in 1000 mL of distilled water with a pH of 7. Each study sample was then placed in 10 mL saliva at room temperature, which was maintained throughout the study to maintain the intraoral temperature.

At each assessment interval, 24 hours, two, four, and six months, samples were kept separately for 5% w/w and 1% w/w titanium dioxide to attain artificial saliva free of impurities as impurities settle at the base of the storage box. The samples were purified before quantification of titanium content and the not-needed-organic-matters are removed. The purification was done using either stabilization, dilution, or centrifugation process. ICP-MS was used to assess the titanium in even trace amounts in the biological fluids.

The data gathered were analyzed statistically using SPSS software version 21.0 (IBM Corp. NY, USA) with t-test and Mann-Whitney U test for intergroup and intragroup comparison, respectively. The p-value with <0.05 was taken as a statistically significant level.

## Results

**Figure 1:**
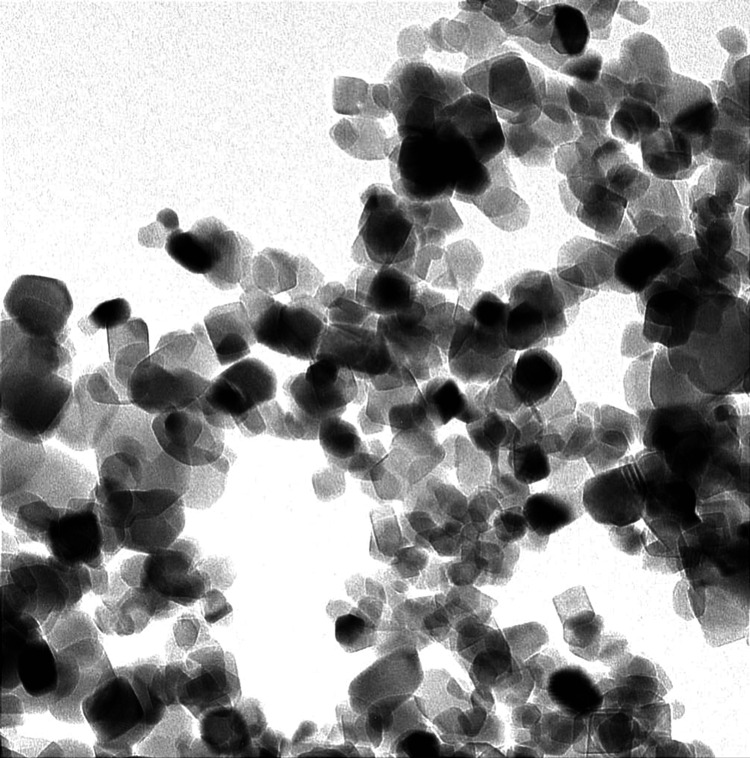
Mean release of titanium from 1% w/w composite having titanium dioxide nanoparticles w/w, weight/weight

**Figure 2:**
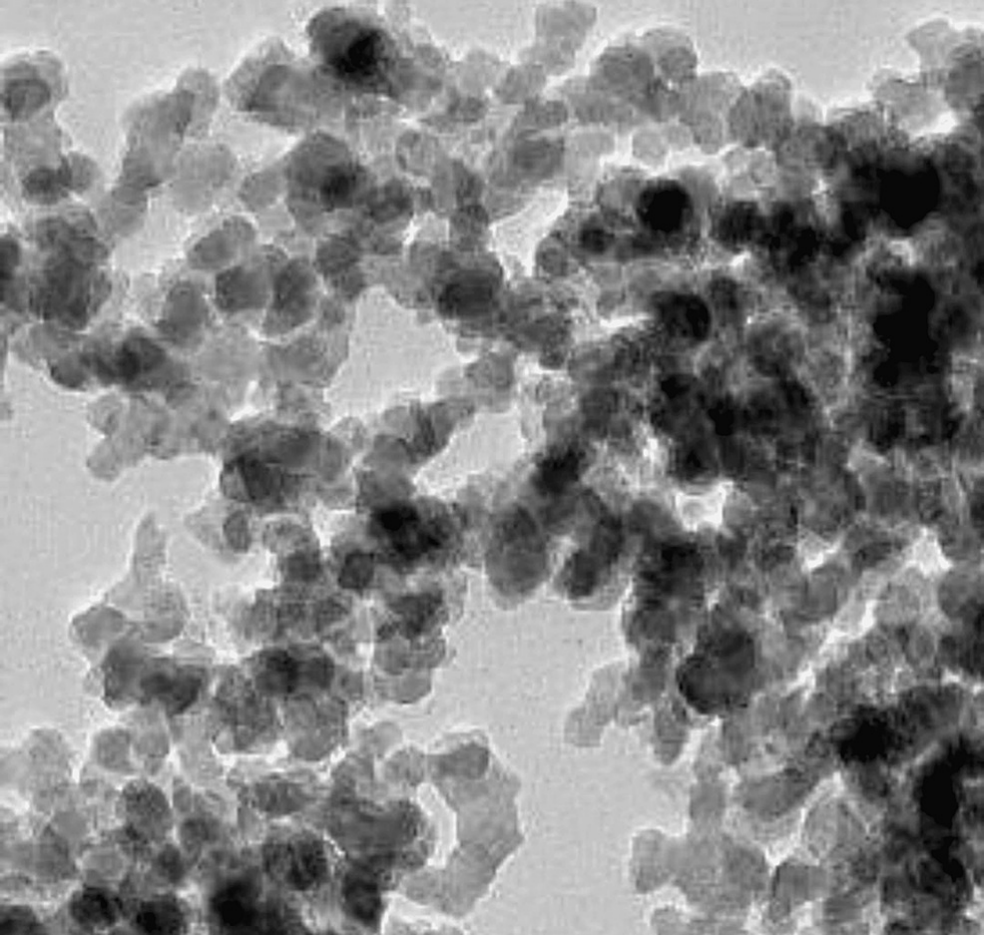
Mean release of titanium from 5% w/w composite having titanium dioxide nanoparticles w/w, weight/weight

The present study assessed 160 premolars extracted for orthodontic purposes that were divided into two groups having 80 teeth each. The selected teeth were bonded to the orthodontic brackets using composite and were impregnated with 5% w/w and 1% w/w nanoparticles of titanium dioxide as shown in Figure 1 and Figure 2 for Groups I and II, respectively. This was followed by the placement of the sample teeth in artificial saliva. The samples were then assessed at 24 hours, two months, four months, and six months.

At 24 hours assessment, the mean release of titanium from 1% w/w composite having titanium dioxide nanoparticles was 0.103±0.007, which decreased significantly to 0.395±0.013 at two months assessment with p=0.001. The mean release of titanium at four months and six months decreased to 0.311±0.013 and 0.223±0.011. This decrease was statistically significant at four and six months with p=0.001, as shown in Table 1.

**Table 1:** Mean release of titanium from 1% w/w composite having titanium dioxide nanoparticles ppb, parts per billion; SD, standard deviation; w/w, weight/weight

Assessment time	Number (n)	Maximum (ppb)	Minimum (ppb)	Mean±SD	p-value
24 hours	20	0.117	0.093	0.103±0.007	0.001
Two months	20	0.413	0.366	0.395±0.013	
Four months	20	0.333	0.294	0.311±0.013	0.001
Six months	20	0.244	0.207	0.223±0.011	

The study results showed that the mean release of titanium from 5% w/w composite having titanium dioxide nanoparticles at 24 hours was 0.048±0.006 that decreased significantly to 0.224±0.365 at two months with p=0.05. A further non-significant decrease was seen at four months and six months to 0.185±0.000 and 0.127±0.135, respectively. The difference was statistically non-significant with p=0.26, as depicted in Table 2. 

**Table 2:** Mean release of titanium from 5% w/w composite having titanium dioxide nanoparticles ppb, parts per billion; SD, standard deviation; w/w, weight/weight

Assessment time	Number (n)	Maximum (ppb)	Minimum (ppb)	Mean±SD	p-value
24 hours	20	0.054	0.036	0.048±0.006	0.05
Two months	20	0.262	0.173	0.224±0.365	
Four months	20	0.185	0.185	0.185±0.000	0.26
Six months	20	0.148	0.105	0.127±0.135	

On comparing the mean release of titanium from 5% w/w and 1% w/w composites having titanium dioxide nanoparticles, it was noted that at 24 hours, mean titanium release was 0.103 and 0.044 from 5% w/w and 1% w/w titanium dioxide nanoparticles that was significantly higher in 5% w/w titanium dioxide with p=0.04. A similar significantly higher titanium dioxide release was seen in 5% w/w titanium dioxide at two months, four months, and six months with p=0.04, 0.001, 0.04, and 0.04, respectively, as shown in Table 3.

**Table 3:** Comparison of the mean release of titanium from 5% w/w and 1% w/w composites having titanium dioxide nanoparticles w/w: weight/weight

Assessment time	Number (n)	5% w/w titanium dioxide nanoparticles	1% w/w titanium dioxide nanoparticles	p-value
24 hours	20	0.103	0.044	0.04
Two months	20	0.395	0.224	0.001
Four months	20	0.311	0.185	0.04
Six months	20	0.223	0.127	0.04

## Discussion

Apart from the orthodontic appliances, plaque retention is also seen on the bonding materials, which can lead to bonding failure in the orthodontic brackets that further delays the treatment, as suggested by Weir E et al. in 2008 [10]. Various material and teeth-related factors can affect the failure rates and bonding systems in orthodontic treatment. Nearly 60-75% of the subjects undergoing fixed orthodontic treatment report enamel demineralization along with bacterial accumulation in small gaps of nearly 10 mm at the junction of enamel and adhesives, as reported by Sukontapatipark W et al. in 2001 [11].

The most commonly used adhesives in orthodontic practice are the composite and the GIC. Despite having the appreciated property of releasing fluoride, GIC has a lesser ability to withstand high forces from the posterior teeth owing to its low fracture toughness, as suggested by Xu HH et al. in 2002 and Hajrassie MK et al. in 2007 [12,13]. On the contrary, composites have the added advantage of being esthetically acceptable, as reported by Aly AA et al. in 2012. Borzabadi-Farhani A et al. in 2014 reported that with their inbuilt roughness, composites tend to gather more plaque and biofilms in the oral cavity compared to other restorative materials [14]. To improve the properties of composites and to combat these shortcomings, resin monomers addition and inorganic fillers pretreatment was suggested by Lu H et al. in 2005 [15]. For reduction of surface roughness, fracture resistance, and antibacterial properties, various components such as titanium dioxide, amphiphilic liquids, tannic acid and derivatives, and/or silver are used. These hybrid composites are used as adhesives to overcome bacterial invasion at the margin of the bracket and to provide better treatment outcomes. 

Nanotechnology, these days, includes matter manipulation atom by atom, which is now being implemented to improve the antibacterial and mechanical properties of orthodontic materials used for bonding purposes. Mechanical properties like fracture toughness and compressive strength can be improved and polymerization shrinkage can be minimized with the compact loading of the nanoparticle fillers. The decrease in the orthodontic adhesive roughness can help in preventing the adhesion of the bacteria. Significant bactericidal activity has been seen with the metal nanoparticles of 1-10 mm, as also confirmed by Allaker RP et al. in 2010 [16].

The most commonly used nanoparticles are titanium dioxide, with different particle sizes based on the use. They have particles of size 0.1 to 0.3 µm known as fine particles, and of size <100 nm considered ultrafine particles. These particles imply the minimum toxicity with antibacterial effects. Titanium dioxide nanoparticles constantly release superoxide ions and hydroxyl radicals on non-lethal ultraviolet light exposure leading to the decomposition of organic compounds reported by Baranowska-Wojcik E et al. in 2020 [17]. Jiang J et al. in 2008 reported that phase and size govern the nanoparticle activity [18]. Nanoparticles of 9 sizes were considered ranging from 4 to 195 nm. The authors reported that below 10 nm and above 30 nm particle size showed constant particle activity, which uniformly decreased from 30 to 10 nm. The highest activity was seen for the anatase and amorphous phase. These findings were consistent with the present in-vitro study that assessed anatase phase particles of sizes 20 to 30 nm, which exposed the possible titanium release activity that relatively depends on both the phase and the size.

Previous studies by Haghi M et al. in 2012 and Sodagar A et al. in 2017 reported that bond strength and antimicrobial properties exist in the titanium-based adhesives with mean bond strength being 13.9±6.00256 and 18.17±4.6564 after the addition of 5% and 1% titanium dioxide [19,20]. The present study also considered 5% and 1% titanium dioxide with consideration of the previous studies where authors reported adequate bond strength and antimicrobial properties at these concentrations of titanium dioxide nanoparticles. With the increase in the concentration of titanium, bond shear strength is reduced, as confirmed by Poosti M et al. in 2013 [21]. Hence, the present study assessed titanium dioxide at 1% and 5% concentrations.

It is vital to assess the permissible levels of titanium dioxide. Previous literature data by Baranowska-Wojcik E et al. in 2020 showed that following oral exposure or inhalation, titanium dioxide nanoparticles aggregate in cardiac muscles, kidneys, spleen, heart, liver, alimentary canal, and lungs, which can further cause intestinal cancer, gastritis, reduced number of microvilli, and spleen and liver damage [17]. Also, neurological and cardiovascular damage has been seen with titanium levels of 10 to 50 mg/kg. In the results of the present study, minimum and maximum concentrations seen were 0.127±0.135 and 0.395±0.013, respectively, which is greatly decreased compared to the levels needed for the mentioned adverse effects in the previous study of Baranowska-Wojcik E et al in 2020 depicting that the concentrations mentioned were within the permissible levels [17].

On comparison of the two concentrations of titanium dioxide at four assessment times, significantly higher quantities were noted in Group I, with 5% titanium dioxide showing a significantly higher titanium release. The decreased release of titanium after two months can be attributed to characteristics of contact inhibition, as confirmed by Metin-Gursoy G et al. in 2017 [22].

The present study was in-vitro in nature and could not simulate the exact intraoral conditions. Also, the diet has a significant impact on titanium release, which was not considered. The sample size taken was lesser owing to its novelty. The study considered the anatase phase; a very less value was seen in the present study compared to the need for adverse effects, which can be attributed to a smaller sample size. However, the study can form the basis of further studies to be conducted in the future where these effects can be further assessed and help to add knowledge to the existing literature.

The present study is an extension to the mentioned article to establish, validate, and confirm the results of the mentioned study in different cohorts of our study.

The study had a few limitations including a smaller sample size, an in-vitro nature, a smaller assessment time, and the use of an artificial salivary medium that could not exactly simulate the ideal intraoral conditions.

## Conclusions

The present study concludes that a higher release of titanium is seen in 5% w/w composite containing titanium dioxide nanoparticles, and the concentrations of 1% and 5% can be safely used and are considered to be within permissible limits. Both 5% and 1% w/w can be used safely in composites. However, at different time intervals, the titanium release might differ. The mean release of titanium dioxide is significantly lesser in 1% titanium dioxide compared to 5% titanium dioxide. Further longitudinal data is warranted to define a definitive conclusion.

## References

[1] Blöcher S, Frankenberger R, Hellak A, Schauseil M, Roggendorf MJ, Korbmacher-Steiner HM (2015). Effect on enamel shear bond strength of adding microsilver and nanosilver particles to the primer of an orthodontic adhesive. BMC Oral Health.

[2] Panchali B, Anam M, Jahirul M, Meryam SR, Ragini M (2016). Nanoparticles and their applications in orthodontics. Adv Dent & Oral Health.

[3] Khatria H, Khajuria A, Gupta P, Jain N (2019). Nano-orthodontics: small is the new big. EC Dent Sci.

[4] Kotrogianni M, Rahiotis C (2017). Resin composites in orthodontic bonding: a clinical guide. J Dent Oral Biol.

[5] Panahandeh N, Torabzadeh H, Aghaee M, Hasani E, Safa S (2018). Effect of incorporation of zinc oxide nanoparticles on mechanical properties of conventional glass ionomer cements. J Conserv Dent.

[6] Eltayeb MK, Ibrahim YE, El Karim IA, Sanhouri NM (2017). Distribution of white spot lesions among orthodontic patients attending teaching institutes in Khartoum. BMC Oral Health.

[7] Mattingly JA, Sauer GJ, Yancey JM, Arnold RR (1983). Enhancement of Streptococcus mutans colonization by direct bonded orthodontic appliances. J Dent Res.

[8] Ruddell DE, Maloney MM, Thompson JY (2002). Effect of novel filler particles on the mechanical and wear properties of dental composites. Dent Mater.

[9] Gutiérrez MF, Malaquias P, Hass V (2017). The role of copper nanoparticles in an etch-and-rinse adhesive on antimicrobial activity, mechanical properties and the durability of resin-dentine interfaces. J Dent.

[10] Weir E, Lawlor A, Whelan A, Regan F (2008). The use of nanoparticles in anti-microbial materials and their characterization. Analyst.

[11] Sukontapatipark W, el-Agroudi MA, Selliseth NJ, Thunold K, Selvig KA (2001). Bacterial colonization associated with fixed orthodontic appliances. A scanning electron microscopy study. Eur J Orthod.

[12] Xu HH, Quinn JB, Smith DT, Antonucci JM, Schumacher GE, Eichmiller FC (2002). Dental resin composites containing silica-fused whiskers—effects of whisker-to-silica ratio on fracture toughness and indentation properties. Biomaterials.

[13] Hajrassie MK, Khier SE (2007). In-vivo and in-vitro comparison of bond strengths of orthodontic brackets bonded to enamel and debonded at various times. Am J Orthod Dentofacial Orthop.

[14] Borzabadi-Farahani A, Borzabadi E, Lynch E (2014). Nanoparticles in orthodontics, a review of antimicrobial and anti-caries applications. Acta Odontol Scand.

[15] Lu H, Stansbury JW, Nie J, Berchtold KA, Bowman CN (2005). Development of highly reactive mono-(meth)acrylates as reactive diluents for dimethacrylate-based dental resin systems. Biomaterials.

[16] Allaker RP (2010). The use of nanoparticles to control oral biofilm formation. J Dent Res.

[17] Baranowska-Wójcik E, Szwajgier D, Oleszczuk P, Winiarska-Mieczan A (2020). Effects of titanium dioxide nanoparticles exposure on human health - a review. Biol Trace Elem Res.

[18] Jiang J, Oberdörster G, Elder A, Gelein R, Mercer P, Biswas P (2008). Does nanoparticle activity depend upon size and crystal phase?. Nanotoxicology.

[19] Haghi M, Hekmastafshar M, Faraz MK (2012). Antibacterial effect of titanium dioxide nanoparticles on the pathogenic strain of E coli. Int J Adv Biotech Res.

[20] Sodagar A, Akhoundi MS, Bahador A, Jalali YF, Behzadi Z, Elhaminejad F, Mirhashemi AH (2017). Effect of TiO2 nanoparticles incorporation on antibacterial properties and shear bond strength of dental composite used in orthodontics. Dental Press J Orthod.

[21] Poosti M, Ramazanzadeh B, Zebarjad M, Javadzadeh P, Naderinasab M, Shakeri MT (2013). Shear bond strength and antibacterial effects of orthodontic composite containing TiO2 nanoparticles. Eur J Orthod.

[22] Metin-Gürsoy G, Taner L, Akca G (2017). Nanosilver coated orthodontic brackets: in vivo antibacterial properties and ion release. Eur J Orthod.

